# Alteration of indicator gut microbiota in patients with chronic sinusitis

**DOI:** 10.1002/iid3.996

**Published:** 2023-09-26

**Authors:** Michał Michalik, Adrianna Podbielska‐Kubera, Anna Maria Basińska, Monika Szewc, Mirosława Gałęcka, Andreas Schwiertz

**Affiliations:** ^1^ MML Medical Center Warsaw Poland; ^2^ Institute of Microecology Poznań Poland; ^3^ Institute of Microecology Herborn Germany

**Keywords:** autoimmunological or allergic symptoms, chronic rhinosinusitis (CRS), immunity, intestinal microbiota, microbiological indicator

## Abstract

**Background:**

Many factors influence the composition of the sinus microflora. The microbial balance is most disturbed by the use of antibacterial agents. Superinfections caused by more than one pathogen may then occur. Despite treatment, including surgery and long‐term antibiotic therapy, many patients with sinusitis do not experience significant relief from their symptoms. It has been hypothesized that an imbalance in the gut microbiota may also be responsible for the chronicity of sinusitis. Our goal was therefore to identify selected gut indicator bacteria that play a role in immunity in patients with chronic sinusitis. In addition, compare the number of selected bacteria in two groups of patients: with chronic sinusitis and with chronic rhinosinusitis (CRS) with concomitant diseases and/or symptoms other than CRS.

**Results:**

Significantly decreased numbers of *Bifidobacterium* spp. and *Faecalibacterium prauznitzi* bacteria were observed in patients from the G1 group. The majority of patients from this group (12 out of 13) had a significantly decreased number of *Bifidobacterium* and *Akkermansia muciniphila* bacteria, which are involved in the nutrition and regeneration of gut epithelium cells and have anti‐inflammatory properties. In group G2 (patients with chronic sinusitis and symptoms of comorbidities) a decreased number of *F. prausnitzii, Bifidobacterium* spp., *A. muciniphila* and *Lactobacillus* spp. bacteria was observed. A small percentage of patients in this group showed overgrowth of yeast‐like fungi.

**Conclusion:**

Although the more research is needed, possibly the gut microbiota indicator bacteria number analyses might enable to plan personalized prebiotic and probiotic treatment, which could support intestine microbiota and mucosal immunity patients suffering from chronic sinusitis.

## Graphical abstract

Alteration of gut microbiota, especially deficiencies of protective bacteria, bacteria nourishing the gut epithelium, and immunostimulant bacteria as well as sinuses microbiota, might be of significance to the processes of healing and regeneration as well as complications after surgical procedures and recurrences of sinusitis. Patients with chronic sinusitis have altered indicator gut microbiota, especially lowered numbers of Bifidobacterium, *Akkermansia muciniphila*, and *Faecalibacterum prauznitz*. In addition, patients with comorbidities also had lowered numbers of Lactobacillus and hydrogen peroxide‐producing Lactobacillus. Deficiencies of the mentioned species and genera might lead to overgrowth of some potential pathogens, for example, *Candida albicans*, *Clostridium difficile*, and Proteus spp.

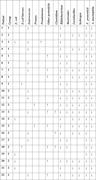

## 1 INTRODUCTION

Sinuses are pneumatic cavities in viscerocranium bones connected to the nasal cavity with narrow passageways. Sinuses include paired frontal, maxillary, sphenoidal, and ethmoid sinuses. Chronic rhinosinusitis causes nasal and sinus mucosal oedema impairing mucosal ciliary movements.^1^, ^2^ The built‐up mucus serves as a nutrient for various bacteria. Conditions inside the sinuses (temperature, humidity, and protection) are ideal for the development of a microbial niche. Within this niche commensal and potential pathogenic strains can proliferate and depending on the conditions this may lead to an overgrowth of harmful bacteria. As a consequence conditions such as inflammations with headaches, facial pain, fever, and tightness sensation in the eye area, which is extremely uncomfortable for patients, can develop.

Sinusitis is one of the most common infectious diseases in developed countries and the incidence is 10%–20%.^1^ Unfortunately, recurrences of sinusitis are rather common, despite pharmacological and endoscopic treatment. The etiology of chronic sinusitis (CRS) is still under investigation. However, many CRS cases are a consequence of unresolved viral infections. The following conditions are conductive to CRS: nasal polyps, allergic reactions, deviated nasal septum, face injuries, respiratory tract infections, hay fever, and other diseases, such as cystic fibrosis, gastroesophageal reflux, HIV, immunological diseases, and exposure to environmental pollution.^3^, ^4^ The majority of these conditions and diseases predispose to CRS recurrences and connect to the function of the immune system, especially the mucosa‐associated lymphoid tissue (MALT).

In the many of CRS viral infections are the primary cause which however do not require antibiotic treatment. The role of bacteria in CRS is unclear. Bacteria located in the sinuses may contribute to the exacerbation of the disease symptoms. The bacteria identified in the sinuses include: *Streptococcus pneumoniae, Haemophilus influenzae, Staphylococcus aureus*, coagulase‐negative staphylococci; Gram‐negative bacteria: *Pseudomonas aeruginosa, Proteus* spp., *Klebsiella* spp., *Enterobacter* spp., *Escherichia coli*, and anaerobic bacteria (*Peptostreptococcus, Prevotella, Porphyromonas*, spp.).^3^ In patients with decreased immunity CRS can also be caused by fungi belonging to the *Mucoraceae* and *Trichocomaenae* families.^5^ It has been showen that many factors influence the sinus microbiota. Besides intrasubject variability, age, and smoking, the composition and distribution of discrete microorganisms is influenced by antibacterial agents. Such agents disrupt the microbial balance and lead to superinfections caused by more than one pathogen.^6^

In clinical practice numerous sinusitis patients do not respond to treatment despite rigorous treatment regimens, including surgical procedures and prolonged antibiotic treatment. To implement effective treatment, it is necessary to properly select the antibiotic based on the antibiogram of bacterial pathogens.

Personalized treatment based on pharmacokinetic and pharmacodynamic properties of medications is necessary. The issue of antibiotic resistance can result from excessive and inappropriate use of antibiotics in CRS patients. A lot of hypotheses explaining the presence of hard‐to‐treat bacterial species in CRS patients have been put forward: formation of bacterial biofilm, intracellular survival of pathogens, and immune response to superantigens of *S. aureus*. So far, none of the hypotheses was confirmed.^7^

Despite fast development of innovative procedural and surgical techniques as well as increasingly better identification of pathogens, causes and systemic factors predisposing a person to chronic sinusitis require further studying. Hence it was hypothesized that an unbalanced immune response may be responsible for chronic rhinosinusitis.^8^

It is generally known that the mucosal surfaces comprise the main port of pathogens entry. Hence, there are a lot of physiological and immunological mechanisms aiming to protect these large areas of the human body. The key mucosal immune elements are the cellular elements (T and B lymphocytes, macrophages, and natural killer cells) and their activity in the mucosa‐associated lymphoid tissue (MALT) and their vast part connected to the gut‐associated lymphoid tissue (GALT). In the Peyer's patches, being a part of the GALT, the immunological training of lymphocytes occurs; in contact with physiological microbiota immune system's cells are activated through cytokine network induction (the Th1/Th2 lymphocytes balance). Then, B lymphocytes evolve into plasma cells that travel through the entire body and take part in the processes of innate immunity through production of secretory immunoglobulin A (sIgA). It has been reported that especially nonpathogenic gut bacteria like *Enterococcus* spp. stimulate the plasma cells and hence induce sIgA on the all mucosa surfaces and thus prevent adhesion of pathogenic bacteria and viruses to epithelia.^9^, ^10^

In the last years it became evident that alterations within the gut microbiota termed dysbiosis can influence the mucosa‐associated immunity and host immunity. It has been suggested that the Th1/Th2 lymphocytes balance might be connected to autoimmune and inflammatory conditions (Th1 predominance) or allergies and infections (Th2 predominance).^11^ Furthermore, a gut microbiota imbalance may play a role in the treatment of patients with chronic sinusitis, especially with coexisting allergies and atopy. Gut dysbiosis, was found in patients with allergies and atopy, autism spectrum disorder, and diseases of affluence, for example, diabetes and cardiovascular diseases.^12^, ^13^, ^14^, ^15^ Moreover the intestinal dysbiosis was observed in patients with gastrointestinal symptoms: irritable bowel syndrome (IBS), constipations, diarrheas, flatulencies or autoimmune disease (Crohn's disease, colitis ulcerosa, rheumatic arthritis).^12^, ^16^, ^17^, ^18^, ^19^

The observed alterations are often characterized by decrease of beneficial and numerous groups of bacteria like lactic acid bacteria for example, *Lactobacillus* spp., *Bifidobacterium* spp. play crucial role in building homeostasis in the gut, especially when antibiotics are used.^20^, ^21^ Furthermore, in older patents witch gastrological symptoms presence in microbiome *Akkermasia muciniphila* and *Faecalibacterium prauznitzi* nourishing gut epithelium^12^, ^17^, ^19^, ^22^ as well as immunostimulant bacteria (*E. coli*, *Enterococcus* spp.) may favors proper immune responses and efficient defense of mucosal barriers also during infections,^18^ also possibly during chronisinusitis.

Aim of the study was to identify selected indicator gut bacteria which play a role immunity in patients with chronic sinusitis. Furthermore, compare numbers of chosen bacteria in two groups of patients: chronic sinusitis patients and CRS patients with comorbidities and/or symptoms other than CRS for example, allergy, atopy, and gastrointestinal symptoms or autoimmune diseases.

We hypothesize that patients with CRS and other symptoms for exampele, allergy, atopy and gastrointestinal symptoms or autoimmune diseases are more susceptible to gut dysbiosis than patients only with CRS. The identification of gut microbiota of alterations may help support standard treatment with targeted microbiota stimulation in laryngological patients in the future.

## 2 MATERIAL AND METHODS

### 2.1 Patient selection

The study included 22 adult patients of the MML Medical Center reporting obstinate recurring, chronic sinusitis, excessive nasal and and/or throat mucus, diminished nasal patency, pain in sinuses for 8–12 weeks, that is, symptoms meeting the definition of chronic sinusitis. ^1^, ^23^ Most of the patients reported even several years of recurring episodes of sinusitis. All patients were informed about diagnostics, potential risks, benefits and alternatives of a given procedure or intervention, as well sigh suitable consents. Patients were previously treated with antibiotics. A precondition for the participation in the study was ending the antibiotic therapy at least a month before the analyses. Patients aged 18–65 years (*n* = 22) were subjected to standard laryngological diagnostics and treatment. The case history was extended, and it included other symptoms and conditions. Based on the results patients were divided into two groups, the first one (G1) consisting of CRS patients (1; *n* = 9) with no other symptoms and conditions, and the second one (G2) consisting of CRS patients (2; *n* = 13) reporting symptoms other than CRS symptoms, for example, flatulence, or with other diagnosed conditions, for example, allergies, atopic dermatitis, rheumatoid arthritis, nonspecific inflammatory bowel diseases, fungal infections, or autism spectrum disorder.

Full description of patients who qualified is presented in Table 1. Moreover, the table includes information on diagnosis and laryngological procedures used during treatment.

**Table 1:** Description of patients who qualified for the study.

No.	Gender	Group	Age	Conditions and symptoms besides CRS	Laryngological treatment	Laryngological diagnosis
1	M	1	58	None	Inferior turbinate coblation, HYDRO	Turbinates hypertrophy
2	W	1	59	None	MIST, HYDRO	Turbinates hypertrophy, deviated nasal septum
3	M	1	65	None	Endoscopic revision of paranasal sinuses, HYDRO	Nasal polyps
4	K	1	56	None	FESS, nasal and sinus polyps removal, HYDRO, microbiology	Turbinates hypertrophy, nasal, and sinus polyps, flaccid soft palate
5	M	1	47	None	FESS, balloon sinuplasty, inferior turbinate coblation, middle turbinate correction	Deviated nasal septum, turbinates hypertrophy
6	M	1	61	None	Inferior turbinate coblation, middle turbinate correction, FESS, HYDRO	Deviated nasal septum, turbinates hypertrophy
7	W	1	24	None	No procedures were conducted	Mucosae dryness, voice weariness, chronic atrophic pharyngitis and laryngitis
8	W	1	62	None	Bilateral middle antrostomy, HYDRO, septoplasty, and nose bridge correction	Deviated nasal septum
9	W	1	32	None	No procedures were conducted	Food intolerances
10	W	2	32	RA, skin pus furuncles, seasonal allergic rhinitis, diarrheas, constipations, flatulencies	FESS + HYDRO + installation of nasal septum obturator, stage I	Paranasal sinusitis
11	M	2	27	Hay fever, allergic rhinitis, asthma	Re‐FESS + inferior turbinate coblation + HYDRO	Turbinates hypertrophy
12	M	2	54	Allergic asthma, flatulencies, bronchitis, upper respiratory tract infections	FESS + HYDRO + multirooted tooth resection with retrograde filling of root canals (SINUS LIFT) + GBR + closed sinus lift	Turbinates hypertrophy
13	M	2	49	Atopic dermatitis, upper respiratory tract infections, flatulencies and constipations, dermatophytosis	HYDRO, soft palate and uvula correction, opening frontal and ethmoid sinuses, nasal and sinus polyps removal, placement of medicinal filling in paranasal sinuses, after a year FESS and HYDRO	Turbinates hypertrophy, nasal and sinus polyps, flaccid soft palate,
14	M	2	65	Lyme disease, sinusitis, allergic rhinitis	FESS + DSN + inferior turbinate coblation + laser removal of nasal cavity adhesions	Turbinates hypertrophy, deviated nasal septum
15	W	2	27	Hives, IBS, diarrheas, genitourinary tract inflammations, upper respiratory tract infections, gastrointestinal candidiasis	No procedures were conducted	
16	M	2	55	Flatulence, dermatophytosis, type 2 diabetes	Septoplasty, inferior turbinate correction, soft palate, and uvula coblation	Turbinates hypertrophy, deviated nasal septum, flaccid soft palate
17	M	2	28	Flatulencies, upper respiratory tract infections, sinusitis	Septoplasty, inferior turbinate decompression, paranasal sinuses revision, bilateral middle antrostomy with lesion removal from paranasal sinuses, HYDRO	Turbinates hypertrophy, deviated nasal septum
18	M	2	18	Autism spectrum disorder	No procedures were conducted	Autism spectrum disorder
19	M	2	34	Crohn's disease, atopic dermatitis, hay fever, flatulencies	Septoplasty, inferior turbinate coblation	Deviated nasal septum, turbinates hypertrophy
20	W	2	34	Flatulencies, tonsillitis, upper respiratory tract infections	No procedures were conducted	Slight throat congestion, lumpy tonsils of irregular shape
21	W	2	29	Genital candidiasis, upper respiratory tract infections, sinusitis, IBS, constipations, diarrheas, acne, flatulencies, cystitis	Endoscopic removal of a cyst in the left maxillary sinus	Sinus cyst
22	M	2	35	Crohn's disease, colitis ulcerosa, flatulencies, diagnosed lactose intolerance	No procedures were conducted	Nasal septum deviation to the right, inferior turbinates hypertrophy, mucus in nasal passages

Abbreviations: CRS, chronic sinusitis; FESS, functional endoscopic sinus surgery; GBR, guided bone regeneration; HYDRO, irrigation of sinuses with the hydrodebrider device; M, man; MIST, minimally invasive sinus technique; No., patient's number; RA, rheumatic arthritis; Re‐FESS, functional endoscopic sinus surgery‐resurgery; RF, radiosurgery, coblation; septoplasty, correction of the deviated septum; W, woman.

### 2.2 Collection of stool samples for microbial analysis

Fresh stool was collected by the patients and send immediately to the laboratory (Instytut Mikroekologii). Following arrival, 0.25 g of faeces was serially diluted in 2.25 mL of 0.85% NaCl. The solution was vortexed for 5 s and in next step serially diluted (to 10^−9^). Then for inoculation 50 μL of the suspension from the specified dilutions were transferred for individual microbiological media. Suspension was spread over the diagnostic media with a sterile loop.

### 2.3 Identification and enumeration microorganisms

The gut microbiota was analyzed by the KyberKompakt PRO test which was developed by the Institut für Mikroökologie, Herborn, Germany (registration No.: D‐ML‐13337‐01‐00). KyberKompakt Pro is a quantitative and qualitative gut microbiota analyses included specially following group of bacteria:

- *Bacteroides* spp.
- *Bifidobacterium* spp.
- *Enterococcus* spp.
- *E. coli* (also *E.coli* the so‐called biovare form—without immunostimulant properties)
- *Clostridium* spp.
- *Lactobacillus* spp. including determination of hydrogen peroxide‐producing *Lactobacillus* spp.,
- *Enterobacteriaceae—Proteus* spp, *Providencia* spp., *Morganella* spp., *Klebsiella* spp., *Enterobacter* spp., *Citrobacter* spp., *Serratia* spp., *Hafnia alvei*, *Pseudomonas* spp.
- *A. muciniphila*
- *F. prausnitzii*
- Quantitative and qualitative determination of yeast
- Quantitative and qualitative determination of mould.

Reference values for the selected genera and species were published elsewhere^12^, ^24^, ^25^, ^26^, ^27^ (Table 2).

**Table 2:** Reference numbers of chosen groups in adults.

	*Escherichia coli*	*E. coli* biovare	*Enterococcus*	*Proteus*	*Pseudomonas*	Other proteolytic bacteria	*Clostridium*	*Bifidobacterium*	*Bacteroides*	*Lactobacillus*	Hydrogen peroxide *Lactobacillus*	*Faecalibacterium prausnitzii*	*Akkermansia muciniphila*
RF	≥10^6^	<2 × 10^4^	≥10^6^	<2 × 10^4^	<2 × 10^4^	<2 × 10^4^	≤10^5^	≥10^8^	≥10^9^	≥10^5^	≥10^5^	≥10^9^	≥10^8^

#### 2.3.1 Identification and enumeration of culturable microorganisms

To culture to culture the individual microorganisms the following media were used:

1. Chromid CPSE agar (BioMerieux): chromogenic medium for evaluation, quantification and direct identification of *E. coli* and isolation of *Proteus* spp., *Pseudomonas* spp. *Enterococcus* spp. and *Klebsiella‐Enterobacter‐Serratia‐Citrobacter* (KESC)
2. Rogosa Agar + TMB + Peroxidase (Heipha): for the isolation of bacteria from the genus *Lactobacillus* (also hydrogen peroxide‐producing *Lactobacillus* spp.)
3. SPM‐Agar with Polymyxin (Heipha): for the isolation of bacteria of the genus *Clostridium* spp.
4. Columbia agar with 5% sheep blood (Becton Dickinson): nonselective, universal medium used for aerobic and anaerobic cultures, undemanding bacteria
5. Enterococcosel agar (Graso): for the isolation and confirmation of cocci of the genus *Enterococcus* spp.

The inoculated SPM medium was immediately placed in anaerostats with a generator anaerobic conditions (GENbox anaer; BioMerieux) and the atmosphere indicator anaerobic and incubated at 37°C ± 2°C for 48 h. Rogosa Agar + TMB + Peroxidase after inoculation was placed in a desiccator with a 15% CO_2_ atmosphere generator (Genbag CO_2_, BioMerieux) and incubated at 37°C ± 2°C for 48 h. Cultured media: CPSE agar, Enterococcosel and Columbia agar were placed under aerobic conditions and incubated at 37°C ± 2°C for 18‐24 h. In case of necessary to identify the cultured bacteria to the species, the biochemical tests were used (GN‐ID Microgen®).

#### 2.3.2 Detection of H_2_O_2_‐production

Following identification, lactobacilli were tested for hydrogen peroxide production as described previously.^28^ Colonies that produced H_2_O_2_ on the agar appeared dark blue or light brown. Colonies, which did not produce H_2_O_2_, were colorless.

#### 2.3.3 Detection of yeasts and molds

To determine numbers of fungi, the 0.25 g of stool sample was incubated (37°C 15 min) with the tripsin and antibiotic coctail (Penicillin/Streptomycin, Sigma‐Aldrich) and next 400 μL of the suspension was transferred to 1.6 mL PBS solution. Then, for inoculation, 100 μL of the suspension were transferred to the medium Sabouraud agar with gentamicin and chloramphenicol (Becton Dickinson). Suspension spread over the medium with a sterile loop. The inoculated medium was incubated at 37°C and 25°C for 48 h in aerobic conditions. In the case of fungal colony growth identification was carried out—yeast‐like fungi were initially identified to species using Candida chromagar chromogenic medium (Becton Dickinson), and mold based on the direct preparation and the mycological key. In cases requiring the use of more accurate identification methods, have been applied RapID™ YEAST PLUS System (Remel).

Aliquots of fecal suspensions were inoculated on two media—Sabouraud Agar with Gentamicin and Chloramphenicol. The cultures were incubated at two different temperatures—25°C and 37°C for 48 h, under aerobic conditions. Yeast‐like fungi colonies were identified on the CHROMagar by color selction of the different Candida species according to the manufacturer. Other fungi colonies were identified by classical mycological methods. In case of necessary to identify the cultured yeast to the species, the biochemical tests RapID™ YEAST PLUS System (Remel) were used.

#### 2.3.4 Molecular identification of microorganisms

Real‐time PCR [qPCR] was used to identify the following species and genera: *F. prausnitzi, A. muciniphila, Bifidobacterium* spp., and *Bacteroides* spp. Bacterial genetic material *was* isolated from stool samples using the QIAamp Fast DNA Stool Mini Kit (QIAGEN). Two hundred micrograms of feces was weighed and put in a sterile test tube. Bacterial DNA was isolated from the stool sample according to the manufacturer's protocol. DNA eluates were frozen and stored for further analyses.

Quantitative PCR amplification and detection were carried out using the primers described in Table 3. PCR amplification and detection was performed using an QABI 7300 device (ThermoFisher Scientific) sequence detection system in optical‐grade 96‐well plates sealed with optical sealing tape. Each reaction mixture (25 μL) was composed of 12.5 μL of QuantiFast SYBER Green PCR Kit (Qiagen), 2.5 μL primer mix (5 µM), 8 μL sterile distilled water, and 2 μL stool DNA.

**Table 3:** Specific primers used to determine numbers of individual bacteria and the total number of bacteria.

Target	Primer name	Primer sequence (5′‐3′)
*Faecalibacterium prausnitzii*	Praus‐F480	CAGCAGCCGCGGTAAA
	Praus‐R631	CTACCTCTGCACTACTCAAGAAA
*Akkermansia muciniphila*	Akk.muc‐F	CAGCACGTGAAGGTGGGGAC
	Akk.muc‐R	CCTTGCGGTTGGCTTCAGAT
*Bifidobacterium* spp.	F‐Bifid09c	CGGGTGAGTAATGCGTGACC
	R‐Bifid06	TGATAGGACGCGACCCCA
*Bacteroides* spp.	Bacter11	CCTWCGATGGATAGGGGTT
	Bacter08	CACGCTACTTGGCTGGTTCAG

For the negative control, 2 μL of sterile distilled water was added to the reaction soluion instead of the template DNA solution. A standard curve was produced using the appropriate reference organism to quantify the qPCR values into number of bacteria/g. The standard curves were prepared in the same PCR assay as for the samples. The fluorescent products were detected in the last step of each cycle. A melting curve analysis was carried out following amplification to distinguish the targeted PCR product from the nontargeted PCR product. The melting curves were obtained by slow heating at temperatures from 55°C to 95°C at a rate of 0.2°C/s, with continuous fluorescence collection. The data was analyzed using the ABI Prism software. Standards used were enumerated using the copy calculation method.^29^

## 3 RESULTS

Samples obtained from all patients (*n* = 22) showed deviations from the standard reference values of the various bacteria. Lower numbers of *F. prausnitzii* were found in 100% of patients (Table 4). Generally, most patients, in both groups, showed lower numbers of *A. muciniphila* (95%), and *Bifidobacterium* (95%), hydrogen peroxide‐producing.

**Table 4:** Comparison of gut microbiota changes in patients.

Patient	Group	*Escherichia coli*	*E. coli* biovare	*Enterococcus*	*Proteus*	*Pseudomonas*	Other proteolytic bacteria	*Clostridium*	*Bifidobacterium*	*Bacteroides*	*Lactobacillus*	hydrogen peroxide *Lactobacillus*	*Faecalibacterium prausnitzii*	*Akkermansia muciniphila*
1	1			↓					↓				↓	↓
2	1								↓		↓	↓	↓	↓
3	1	↓		↓		↑			↓				↓	↓
4	1			↓					↓		↓	↓	↓	↓
5	1						↑		↓	↓			↓	↓
6	1			↓					↓	↓		↓	↓	↓
7	1			↓			↑		↓	↓	↓	↓	↓	↓
8	1						↑		↓		↓	↓	↓	↓
9	1		↑						↓	↓			↓	↓
10	2			↓					↓	↓	↓	↓	↓	↓
11	2			↓					↓	↓	↓	↓	↓	↓
12	2				↑		↑		↓	↓	↓	↓	↓	↓
13	2			↓			↑	↑	↓	↓	↓	↓	↓	↓
14	2	↓							↓		↓	↓	↓	↓
15	2				↑		↑		↓		↓	↓	↓	↓
16	2			↓				↑	↓		↓	↓	↓	
17	2			↓					↓	↓			↓	↓
18	2		↑				↑	↑	↓	↓			↓	↓
19	2	↓											↓	↓
20	2	↓					↑		↓	↓	↓	↓	↓	↓
21	2	↓							↓		↓	↓	↓	↓
22	2			↓			↑		↓		↓	↓	↓	↓

Abbreviations: ↓, indicates numbers significantly below the reference value; ↑, indicates numbers significantly over the reference values (RF–Table 2).

*Lactobacillus* (71%), *Lactobacillus* (67%), and *Bacteroides* (50%) (Table 4). Furthermore, in some patients in both group number of immunostimulanting bacteria: *Enterococcus* spp. (50%) was decreased. In group 2 the lower number of *E. coli* (23%) was detected. In addition, in 41% of the patients an overgrowth of proteolytic bacteria was observed (Table 4).

The first group (G1) consisted of CRS patients with no other symptoms and conditions. All nine patients within this group had significantly lower numbers of *Bifidobacterium*, *A. muciniphila*, and *F. prausnitzii*.

Moreover, in five patients lower number of *Enterococcus* and hydrogen peroxide‐producing *Lactobacillus* was found (Table 4). In group G2 four patients had lower numbers of *Bacteroides*, wheras in group G2—seven patients. We recognize the lower numbers of *Lactobacillus* were in four patients. Whereas three patients had increased numbers of proteolytic bacteria, and in one patient *E. coli* deficiency was observed.

Yeast or fungal overgrowth was detected in seven patients (more than 1 × 10^3^). Identified species included: *Candida albicans, Candida glabrata, Candida zeylanoides*, and *Geotrichum candidum*.

The second group (G2) consisted of 13 CS patients showing symptoms and conditions other than CRS. All patients had lower numbers of *F. prausnitzii*. The majority of the analyzed samples (12 out of 13) showed lower numbers of *Bifidobacterium spp*. and *A. muciniphila*. In 9 out of 13 patients lower numbers of *Lactobacillus* spp. and hydrogen peroxide‐producing *Lactobacilli* were found. While seven patients had lower numbers of *Bacteroidetes spp., six—lower numbers of Enterococcus, and four—lower numbers of E. coli* (Table 4). In addition, elevated/increased numbers of proteolytic bacteria were found in 6 patients. Several patients showed overgrowth of *Proteus* spp. (2/13), *Clostridum* spp. (3/13), yeast (3/13), and mould (2/13).

## 4 DISCUSSION

Alteration of intestinal microbiota weakening the gut‐associated lymphoid tissue (GALT) and mucosa‐associated lymphoid tissue (MALT) function might possibly lead to deficiency of immune response for example, less effective pathogen protection.^24^ The consequence of altered gut microbiota is lower effectiveness of sinusitis treatment, and slow regeneration after surgery. Gut microbiota plays a crucial role in immune system's development at the beginning of human's life; it also influences homeostasis, immune tolerance, and pro‐inflammatory and anti‐inflammatory cytokines production in the adulthood.^16^ On the other hand we expected that the CRS patients treatment may disturb their intestinal microbiota. As dysbiosis is often connected with many diseases, changes in gut microbiota observed in group 2 (G2) are stronger. Sinusitis treatment includes pharmacotherapy and surgical procedures. Pharmacotherapy includes vasoconstrictor drugs, mucolytics, mucus thinners, antipyretics, and antibiotics. In cases of difficulty in draining the mucus form the sinuses due to abnormal anatomy of nasal cavities and sinuses passageways surgical procedures are necessary. Usually functional endoscopic sinus surgery (FESS) is conducted.^30^ The FESS technique allows to restore patency of the natural passageways connecting sinuses and nasal cavity, that is, the so‐called ostiomeatal complexes, and restore a normal ventilation of sinuses. The FESS technique is minimally invasive and precise; it ensures shortened recovery period and reduces surgical intervention in the affected tissues. Risk of scarring is very low. Beside FESS, there are many other method for chronic sinusitis treatment for example: Sinus lavage with the Hydrodebrider method, Balloon sinuplasty, MIST (Minimally Invasive Sinus Technique), CYCLONE sinus wash.

Surgical treatment is usually supported with antibiotic treatment. The empiric antibiotic treatment aims at the most common Gram‐positive microorganisms. Thus, the presence of Gram‐negative pathogens may make the treatment ineffective and/or lengthy.^2^ In the case of coagulase‐negative staphylococci (CNS) the following drugs are used: macrolides, lincosamides, and streptogramin B.^31^ Because historically CNS are considered commensal organisms, the diagnostic process is too often stopped at identification of bacterial genus. The high CNS occurrence not only significantly decreases therapeutic possibilities but it also makes it possible for the resistance mechanisms to be transferred to other staphylococci strains occurring in hospital environment, including virulent pathogens, such as *S. aureus*. CNS strains (except for *S. epidermidis*) isolated from patients treated in the MML Medical Center had constitutive mechanism of resistance to macrolides, lincosamides, and streptogramin B MLSB (9%), macrolids and streptogramin B MSB (15%), and methicillin (9%). Moreover, strains resistant to clindamycin comprised 8% of CNS strains, and strains resistant to aminoglycosides—13%.^32^ It needs to be emphasized that selecting antibiotics based on appropriate microbiological tests and antibiograms with simultaneous diagnostics and potential surgical corrections of the affected sinuses and nasal septum seems to be crucial for effective treatment of patients with both recurring and chronic sinusitis. During treatment of CRS antibiotics and other pharmaceutical treatment has been used. Although its often necessary especially during surgeries, this may decrease an beneficial sinus and gut microbiota species and groups. Especially protective microbiota for example, lactic acid bacteria and species nourishing the gut epithelium intestinal microbiota deficiencies, might be of significance to the processes of healing and regeneration as well as complications after surgical procedures and recurrences of sinuses conditions. The reason for the ineffectiveness of antibiotic treatments is often the fact that microorganisms create a biofilm in the sinuses—a protective layer limiting antibiotics penetration and activity. Inside of the biofilm antibiotics can be inactivated. Bacterial biofilms are conductive to recurrences of chronic sinusitis. Antibiotic treatments, surgical procedures and hospitalization have an impact on gut microbiota and could have affected on dysbiosis observed in both group of patients.

It has been showed that immune system activation by some gut microbiota bacteria is chemically mediated through cells's surface receptors, that is, PRR (pattern‐recognition receptors), in particular the Toll‐like receptors (TLR). TLR bond with certain proteins of pathogenic viruses, bacteria, and own proteins; some of the receptors are still under investigation. The role of these receptors includes identification of pathogenic factors and differentiation between self‐antigens and non‐self‐antigens.^33^, ^34^ Whereas, the Toll‐like type 4 receptors (TLR4) bond with fragments of gram‐negative bacteria, for example, *E. coli*, in particular with lipopolysaccharides (LPS). As the TLR4 are present in the immune system's cells (macrophages, dendritic cells, mast cells, eosinophiles, neutrophiles, and B lymphocytes) the LPS activates all of these cells.^18^, ^35^, ^36^ This causes production of antibacterial factors and pro‐inflammatory cytokines as well as maturation of dendritic cells (increased expression of the co‐stimulating molecules and MHC) that have the ability to present antigens. Activation of antigen‐presenting cells by TLR, among other things, results in increased synthesis of pro‐inflammatory cytokines (TNF‐a, IL‐1, −6, −8, −12) and chemokines, and expression of adhesion and co‐stimulating molecules (CD40, CD80, CD86). TLR receptors directly or indirectly influence the activity of the regulatory cells Treg CD4+CD25+ by inhibiting the immune response or waiving their activity with suppression.^18^, ^34^ Immunostimulant bacteria are responsible for keeping cytokine balance and stimulating lymphocytes to adequately react to bacterial and viral antigens. For instance, nonpathogenic cocci of the *Enterococcus* genus stimulate plasma cells to synthetize secretory immunoglobulin A (sIgA) on all mucosae. Immunoglobulins sIgA are the first line of defense against pathogenic bacteria and they show bacteriostatic properties, neutralize bacterial toxins, coat pathogenic microorganisms, and prevent their penetration into the body. Observed in several patients from both groups lowered numbers of immunostimulant *Enterococcus* and *E. coli* bacteria could have additionally intensified immunity deficiencies and could have been conductive to recurrence of upper respiratory tract infections. Deficiencies of sIgA at mucosal surfaces increase the risk of infections caused by skin and mucosal microbiota, for instance, the coagulase‐negative staphylococci (CNS). CNS are often isolated from patients suffering from maxillary sinusitis and according to the American Academy of Otolaryngology‐Head and Neck Surgery Multidisciplinary Rhinosinusitis Task Force, CNS comprise the second etiologic factor of chronic sinusitis.^37^

In the study, significant alterations of gut microbiota were found in all of the CRS patients (G1, G2) despite other conditions and symptoms not related to sinuses function. All of the patients of the group G1 had significantly lowered numbers of *Bifidobacterium* bacteria and in all the samples lowered numbers of *F. prauznitzi* were also found. Majority of the patients from this group (12 of 13) had severely lowered numbers of *Bifidobacterium* and *A. muciniphila*. *A. muciniphila* and *F. prauznitzi* cooperate in the production of short‐chain fatty acids (SCFA), especially the butyric acid. It is a very important compound nourishing and regenerating gut epithelium cells. Moreover, it has anti‐inflammatory properties what seems to be significant in patients with nonspecific inflammatory bowel diseases and constipations.^17^, ^38^, ^39^, ^40^ Patients with Crohn's disease, besides lowered numbers of *F. prauznitzi*, also had lowered amounts of butyric acid in stool.^17^, ^19^

*Bifidobacterium* spp. are anaerobic gram‐positive bacteria of variable cell shapes, from irregular cocci to ramified Y‐shape cells, commonly found in human, animal, and insect digestive systems. These are one of the first bacteria populating digestive system and irregular numbers of these bacteria seems to be directly connected to health issues of the host. *Bifidobacterium* spp. is one of the most abundant bacteria in human gut (ca. 10^9^ colony forming units/g of stool) and produce acetic and propionic acid, vitamin B, and folic acid; they also inhibit proliferation of pathogens and restore microbiological balance after antibiotic treatment.^24^, ^41^ Studies emphasize that these are the most common pro‐health properties of *Bifidobacterium* bacteria species and because of that they are used as probiotic bacteria.^41^ Studies proved the highest health‐related benefits of *Bifidobacterium infantis*, *Bifidobacterium lactis* and *Bifidobacterium breve* strains. Their properties included improving intestinal passage, improving immunity, taking part in the production of SCFA, and improving symptoms of diarrhea, irritable bowel syndrome, and nonspecific inflammatory bowel diseases.^41^

Besides *Bifidobacterium*, protective bacteria include *Bacteroides* and *Lactobacillus*, also abundant in the gut. As many as 9 of 13 patients of the group G2 had lowered levels of *Lactobacillus*, and hydrogen peroxide‐producing *Lactobacillus*. Whereas in seven patients lowered numbers of *Bacteroides* spp. were found. Other conditions and symptoms, for example, allergies, flatulencies, atopic dermatitis, autoimmune conditions, or IBS diagnosed in patients in addition to the sinusitis according to literature can be connected with abnormal gut microbiota.^41^ Microbiota abnormalities were observed in patients suffering from atopic conditions, inflammatory bowel diseases, Crohn's disease, ulcerative colitis, infectious colitis, and diabetes multiple times.^41^ In a group of patients suffering only from the sinusitis lowered numbers of *Bacteroides* and *Lactobacillus* were observed less frequently. One of the extensively described properties of protective bacteria, especially *Bifidobacterium* and *Lactobacillus*, is production of lactic acid acidifying gut contents what inhibits the development of pathogenic bacteria and fungi. Moreover, proper numbers of these bacteria in the gut favors microbiological balance and supersede pathogenic bacteria and fungi thanks to taking up the receptors adhesion sites on the gut epithelium (competition for space) and using the available resources (nutrient competition).

Some *Lactobacillus* species, for example, *L. acidophillus*, produce bacteriocins and hydrogen peroxide that inhibit growth of other bacteria. Thus, taking antibiotics and improper diet can contribute to deficiencies of these bacteria and to the release of ecological niche for the potentially pathogenic bacteria, for example, *Clostridium difficile*, and fungi, for example, *C. albicans*. In the group G1 seven patients had overgrowth of yeast in the stool at the temperature of 37°C. This included *C. albicans, C. glabrata, C. zeylanoides*, and *G. candidum*. These species can colonize digestive tract, however, their excess can cause pain, flatulence, and diarrhea Elevated numbers of proteolytic bacteria was observed in stool samples obtained from six patients of the group G2. In individual patients overgrowth of *Proteus* spp. (2/13), *C. difficile*, yeast‐like fungi (3/13), and mould fungi (2/13).

Proteolytic bacteria are one of the potentially pathogenic microorganisms and their overgrowths can cause flatulencies, odorous gases and diarrhea. Toxicogenic *C. difficile* strains are the main pathogen responsible for the acute *C. difficile*‐associated diarrhea. Patients with recurring and chronic sinusitis are also a group at risk of the antibiotic‐associated diarrhea, prevention of which includes supplementation of protective strains of *Lactobacillus rhamnosus* GG and *Sacharomyces boulardi*, which was confirmed in a metanalysis of clinical studies.^42^, ^43^

In the case of the studied groups many patients showed factors predisposing them to CRS. This included morphological defects hindering proper cleanse and ventilation of sinuses and their passageways as well as the nasal passage, for example, septum deviation, nasal polyps. Besides this factor also abnormal gut microbiota observed in both of group is a strong risk factor of infection. The following conditions predispose patients to CRS to a similar extent: conditions connected to excessive mucus production (hay fever, airborne allergies), atopic dermatitis, allergic reactions, asthma, and respiratory tract infections. This analysis confirmed the factors reported in the literature.^3^, ^4^, ^44^ Autoimmune diseases are also mentioned as one of the factors predisposing to CRS.^3^, ^4^ Numerous antibiotic treatments used to treat sinusitis undoubtedly decreased gut bacteria numbers, despite the fact that for at least a month before the study patients had not used antibiotics. This supports observations of other authors that taking antibiotics even for a short time in the course of upper respiratory tract infections disrupts numbers of many species for a long time, and restoring previous state can take up to 4 years.^20^ Moreover, new studies suggest that taking antibiotics, but also other nonantibiotic drugs, can inhibit the development of from several to a few dozen gut microbiota species and decrease their numbers.^21^ Therefore, it would be beneficial to strengthen gut microbiota in patients with recurring infections, in addition to standard treatment. Such a solution should be also considered in patients with gut barrier disorders, that is, patients suffering from autoimmune diseases, liver diseases, and metabolic disorders as well as older patients.

### 4.1 Potential and safe methods of strengthening gut microbiota in CRS patients

Many reports indicated immunostimulant properties of some bacterial particles, many vaccines, bacterial lysates (killed cells), and live probiotic strains.^36^, ^42^, ^43^, ^45^ Two metanalyses reported lowered average numbers of respiratory tract infections and lowered frequency of using antibiotic treatments in children taking bacterial lysates in comparison to placebo groups.^36^, ^46^, ^47^

Clinical research showed that properties of probiotics depend on used strain and are related to certain thoroughly studied groups.^42^, ^43^ Effectiveness of some probiotics, for example, the *Enterococcus faecalis* DSM 16440 strain, was proven in children and adults also with recurring respiratory tract infections. Decreased average numbers of recurrences and frequency of administering antibiotics were observed in patients supplemented with *E. faecalis* DSM 16440 in comparison to a group taking placebo.^48^ MultiCenter randomized, double‐blind studies conducted on 157 patients showed effectiveness of the *E. faecalis* DSM 16440 strain supplement in decreasing the frequency of recurrences in patients with chronic recurring hyperplastic sinusitis. Number of recurrences in the studied group (50 cases) was lower by half (50%) in comparison to the number observed in the group taking placebo −90.^49^ In a majority of studies it was possible to start the treatment during standard antibiotic treatment, no side effects were noted and safety of used preparations was proven.

Even larger number of studies proved pro‐health properties of probiotics containing the *Lactobacillus* and *Bifidobacterium* protective bacteria strains. However, when modulating microbiota, one should always remember to use high‐quality probiotics containing safe and accurately described and tested strains.^41^, ^42^, ^50^

Regarding *A. muciniphila* and *F. prauznitzi*, theirs beneficial impact on human health was proven but the clinical application of *A. muciniphila* and *F. praustnitzi* due to limitations in culture conditions is very limited. However, their growth can be stimulated with dietary resistant starch and, to a lower extent, inulin (prebiotics). Food products rich in these prebiotics include chilled starch products, rolled oats, chicory, asparagus, and unripe bananas.^50^ It is worth noticing that vegetables, unprocessed and whole‐grain foods, nuts, seeds, and fruits are considered foods beneficial for gut microbiota.^51^

Observed in all patients imbalance in gut microbiota could be a reason of chronic sinusitis of these patients, therefore the further research focusing on improvment of microbiota patients with chronic sinusitis are needed.

## 5 CONCLUSIONS

Alteration of gut microbiota, especially deficiencies of protective bacteria, bacteria nourishing the gut epithelium, and immunostimulant bacteria as well as sinuses microbiota, might be of significance to the processes of healing and regeneration as well as complications after surgical procedures and recurrences of sinusitis. We found that patients with chronic sinusitis have altered indicator gut microbiota, especially lowered numbers of *Bifidobacterium, A. muciniphila*, and *F. prauznitz*. In addition, patients with comorbidities also had lowered numbers of *Lactobacillus* and hydrogen peroxide‐producing *Lactobacillus*. Deficiencies of the mentioned species and genera might lead to overgrowth of some potential pathogens, for example, *C. albicans*, *C. difficile*, and *Proteus* spp.

Strengthening gut microbiota ensures proper function of gut microbiota and its barrier; may protects from pathogenic viruses and bacteria thanks to stimulation of certain immunological mechanisms. Determination of gut microbiota indicator bacteria numbers might enable to plan more personalized targeted probiotic treatment that might be helpful in supporting the treatment of patients suffering from chronic sinusitis. It seems necessary to conduct further studies on the effectiveness of probiotic therapy strengthening the chosen gut microbiota species in conditions related to dysbiosis, including chronic and/or recurrent infections like sinusitis.

## AUTHOR CONTRIBUTIONS

**Michał Michalik**: Conceptualization; methodology; resources; supervision; writing—original draft; writing—review & editing. **Adrianna Podbielska‐Kubera**: Visualization; writing—review & editing. **Anna Maria Basińska**: Conceptualization; investigation; methodology; resources; supervision; visualization; writing—original draft; writing—review & editing. **Monika Szewc**: Investigation; methodology; supervision; writing—original draft; writing—review & editing. **Mirosława Gałęcka**: Conceptualization; investigation; methodology; resources; supervision. **Andreas Schwiertz**: Supervision; writing—review & editing.

## CONFLICT OF INTEREST STATEMENT

The authors declare no conflict of interest.

## ETHICS STATEMENT

Written informed consent has been obtained from the patient(s) to publish this paper.
